# Topically Applied Cross-Linked Hyaluronan Attenuates the Formation of Spinal Epidural Fibrosis in a Swine Model of Laminectomy

**DOI:** 10.1038/s41598-019-50882-x

**Published:** 2019-10-10

**Authors:** Cheng-Li Lin, I-Ming Jou, Cheng-Yi Wu, Yuh-Ruey Kuo, Shih-Chieh Yang, Jung-Shun Lee, Yuan-Kun Tu, Sung-Ching Chen, Yi-Hung Huang

**Affiliations:** 1 Department of Orthopaedic Surgery, National Cheng Kung University Hospital, College of Medicine, National Cheng Kung University, Tainan, Taiwan; 2 Skeleton Materials and Bio-compatibility Core Lab, Research Center of Clinical Medicine, National Cheng Kung University Hospital, College of Medicine, National Cheng Kung University, Tainan, Taiwan; 3 Department of Orthopedics, E-Da Hospital, I-Shou University, Kaohsiung, Taiwan; 4 Department of Orthopedics, Chia Yi Christian Hospital, Chia Yi, Taiwan; 5 Division of Neurosurgery, Department of Surgery, National Cheng Kung University Hospital, Tainan, Taiwan; 6 Maxigen Biotech Inc., Taoyuan, Taiwan; 7 Department of sports management, Chia Nan University of Pharmacy & Science, Tainan, Taiwan

**Keywords:** Neurosurgery, Preclinical research

## Abstract

Epidural fibrosis is an inevitable aspect of the postoperative healing process which is one of the causes of failed back surgery syndrome following spinal surgery. The aim of the present study was to examine the inhibitory effect of 1,4-butanediol diglycidyl ether-crosslinked hyaluronan (cHA) on spinal epidural fibrosis in a swine model. Epidural fibrosis was induced through conduction of hemi-laminotomy (L2 and L3) or laminectomy (L4 and L5), while L1 was assigned as the control group in six pigs. The cHA was applied to L3 and L5 surgical sites. MRI evaluation, histologic examination, expressions of matrix metalloproteinases (MMPs), and cytokines in scar tissue were assessed four months after surgery. cHA treatment significantly decreased the scar formation in both hemi-laminotomy and laminectomy sites. cHA also significantly increased MMP-3 and MMP-9 expression in scar tissue. Further, the epithelial-mesenchymal transition -related factors (transforming growth factor-*β* and vimentin) were suppressed and the anti-inflammatory cytokines (CD44 and interleukin-6) were increasingly expressed in cHA-treated sites. The current study demonstrated that cHA may attenuate spinal epidural fibrosis formation after laminectomy surgery by enhancing the expression of MMPs and anti-inflammatory pathways.

## Introduction

Epidural fibrosis (EF) is a regenerative process, involving the formation of extradural fibrous tissue after spinal surgery. EF may tether nerve roots to adjacent tissues, restricting the mobility of the nerve roots as well as increasing tension on the nerve roots during motion; ultimately, this can lead to nerve injury and recurrent radicular pain^1,2^. EF on surgically exposed dura mater and on nerve roots is one of the factors leading to suboptimal outcomes after spine surgery^1–3^. Numerous biomaterials have been developed to prevent postoperative EFs^4–9^.

Hyaluronic acid (HA) is an anti-adhesive and anti-fibrotic agent that has been used to inhibit peripheral nerve adhesion, scar formation, and fibrosis formation after experimental laminectomy^4,5^. Crosslinking of linker molecules, such as divinyl sulfone, homobifunctional glycidyl ethers, glutaraldehyde, and formaldehyde with polymer chains of HA improves the mechanical properties and lengthens the residence time^8–10^. However, these linker molecules are often toxic, irritative, or corrosive and can potentially be released into surrounding tissues during the degradation of crosslinked hyaluronic acid. 1,4-butanediol diglycidyl ether (BDDE)-crosslinked hyaluronan (cHA) is synthesized by a novel cross-linking process. It is a non-resorbable cross-linked hyaluronan-derived polymer with minimal cytotoxicity and adverse effects in regards to electrophysiology and neurobehavior^8,10^. The effectiveness of topically applied cHA in the reduction of EF in rats has been demonstrated by Wu and colleagues^6^.

The matrix metalloproteinases (MMPs) are responsible for the remodeling of connective tissues under normal physiologic and pathologic conditions. The MMPs play important roles in regulating extracellular matrix (ECM) turnover in fibrotic tissues^11,12^. Degradation of normal ECM components in the early stages of fibrosis during repair and scar formation promotes the deposition of newly synthesized collagen through cell migration mediation, leukocyte activation, antimicrobial defense mechanisms, and inflammatory reactions^13–17^. The aim of the present study was to determine the functional effects of cHA hydrogels on epidural fibrosis in a porcine model of spinal laminectomy as cHA hydrogels have been recognized as biophysical and chemical modulators of tissue fibrosis. In addition to histological assessments, this study will also attempt to describe the mechanism of cHA hydrogels’ anti-fibrosis effects. It was hypothesized that the 1,4-butanediol diglycidyl ether-crosslinked hyaluronan would have inhibitory effects on EF through the effects of MMPs-mediated ECM remodeling and anti- inflammatory pathways.

## Results

All the six Lanyu Small-Ear Pigs were in health status and no adverse events were identified during the whole experimental period. All the pigs (6/6) were used in the data analysis.

### cHA attenuated the formation of epidural fibrosis after surgical intervention

**Figure 1:**
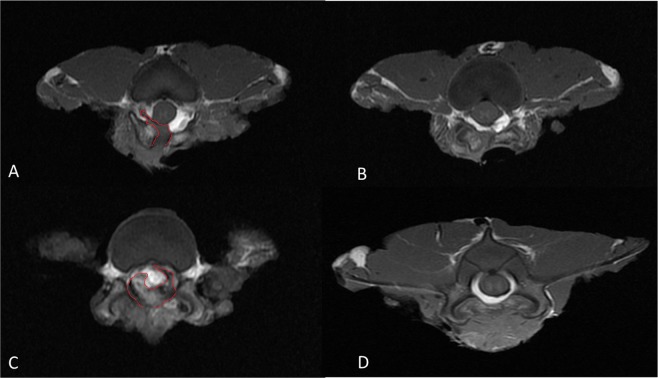
The extent of EF was less in the cHA group (**A**,**C**) than the non-cHA group (**B**,**D**).

At postoperative week 16, MRI was performed and images were analyzed using the Ross grading system. Compared with Hemi and Lami groups, significantly less tissue fibrosis were observed in the epidural region of Hemi-HA and Lami-HA groups, respectively (p < 0.05) (Fig. 1, Table 1). Treatment with cHA significantly reduced the proportions of high grading (grades 3 and 4) of epidural fibrosis in the Hemi and Lami groups (10% vs 26.6% and 3.3% vs 8.4%, respectively (Table 1). Besides, the degrees of EF was consistently greater in laminectomy than hemi-laminotomy groups, despite of the treatment with cHA (P < 0.05). Therefore, the tissues harvested from the laminectomy groups were used for the subsequent molecular and biochemical analysis.

**Table 1:** Radiological scores using the Ross grading system.

Groups				
Grade	Hemi	Hemi-HA	Lami	Lami-HA
1	35%	59.2%	0%	27.5%
2	36.7%	31.7%	18.3%	51.7%
3	18.3%	5.8%	55%	12.5%
4	8.3%	3.3%	23.3%	6.7%
5	1.7%	0%	3.3%	1.7%
	*p* < 0.05		*p* < 0.05	

### cHA potentiated the organization of ECM with increased cell proliferation and decreased programmed cell death

**Figure 2:**
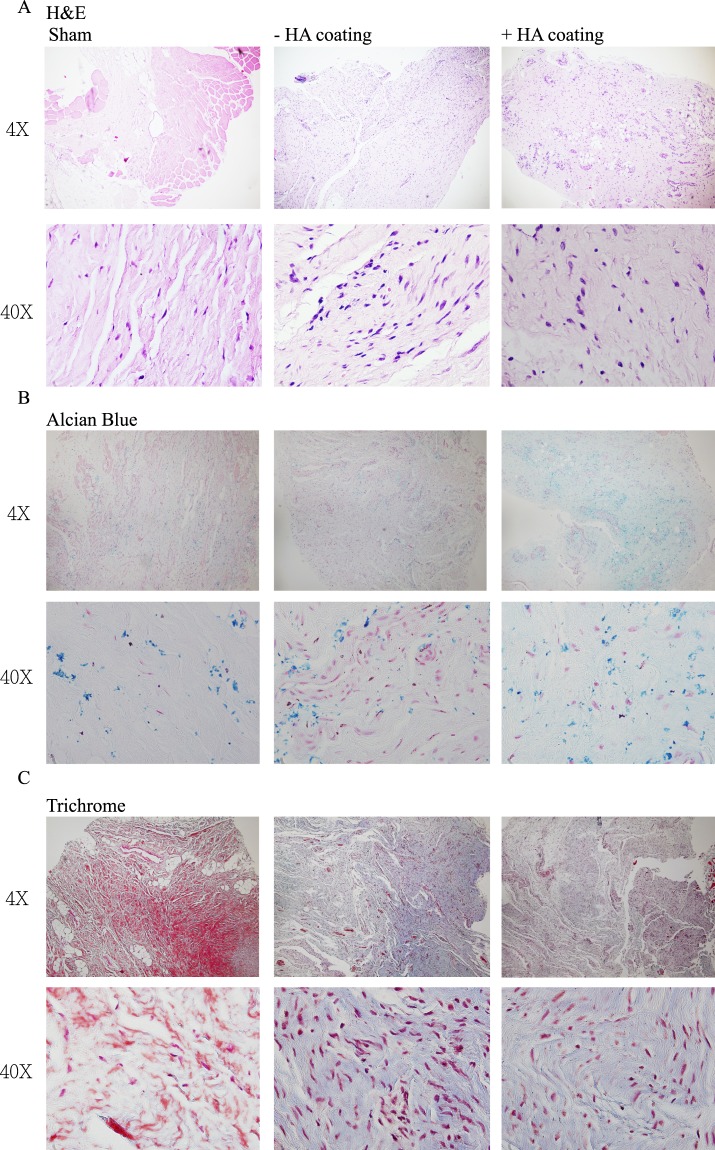
(**A**) H&E staining showed better organization of the ECM in the cHA group. (**B**) Alcian blue staining showed the HA expression in the cHA groups was similar with control groups and was enhanced compared to the non-cHA groups. (**C**) Trichrome staining showed less collagen and elastic fibers deposition in the cHA group.

H&E staining showed better organization of the ECM in the remodeled tissue with reduced infiltration of inflammatory cells in the cHA group that was similar to the levels of control groups (Fig. 2A). Figure 2B showed that the HA expression in cHA groups was not different from the controls, and was enhanced in comparison to the non-cHA groups by Alcian blue staining. Trichrome staining illustrated less collagen and elastic fibers were deposited in the epidural tissue of cHA-treated group (Fig. 2C).

**Figure 3:**
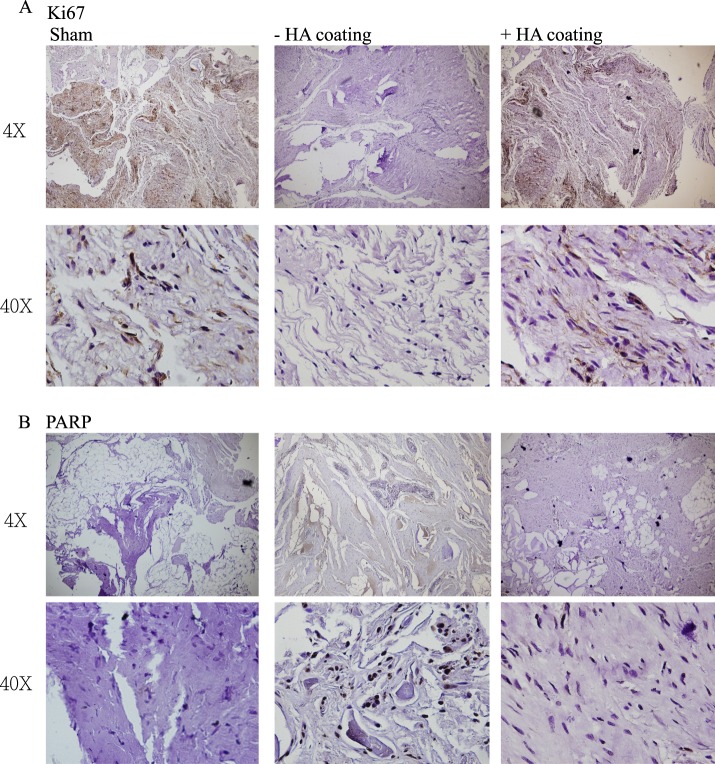
(**A**) Increased Ki-67 expression was demonstrated in the cHA group. (**B**) PARP expression was suppressed in the cHA group.

Increased expressions of Ki-67 were observed in the cHA-treated biopsies than those observed in the non-cHA group (Fig. 3). In addition, expression of poly (ADP-ribose) polymerase (PARP) in the epidural tissues was highly suppressed in cHA group, suggesting the reduction in cell apoptosis (Fig. 3B).

### cHA enhanced the expression of MMPs in the epidural tissues

**Figure 4:**
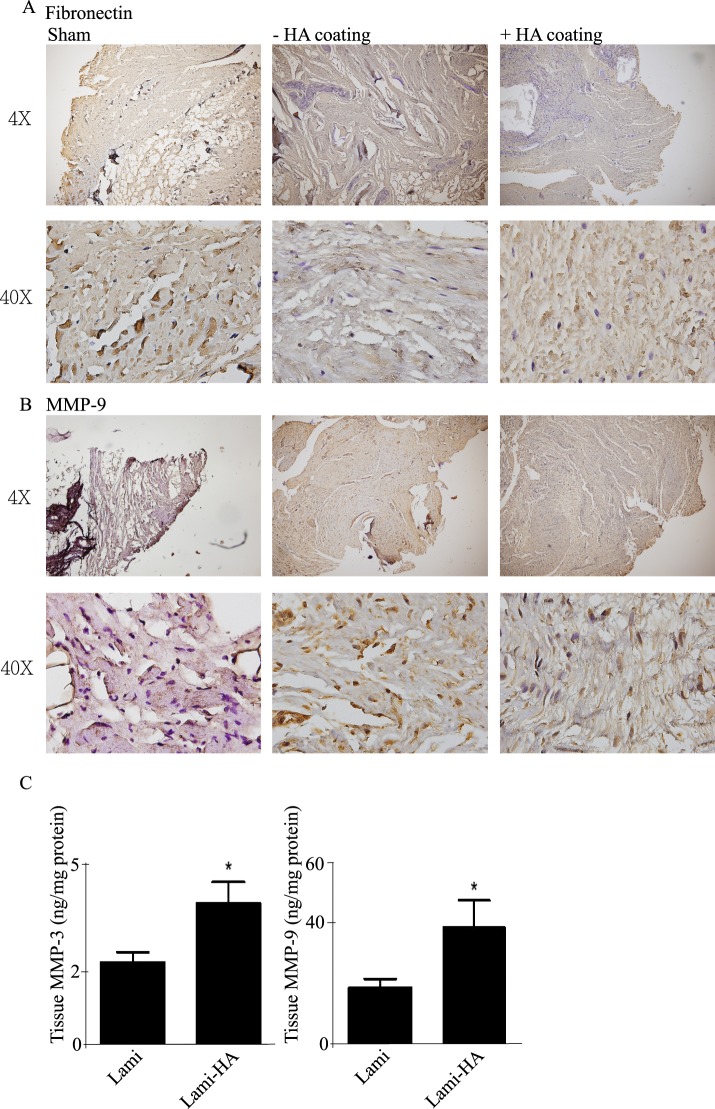
(**A**) There was no significant difference in fibronectin levels in each group. (**B**) The expression of MMP9 was enhanced in the cHA group. (**C**) MMP3, and 9 levels were both up-regulated in the cHA groups compared with the non-cHA treated groups.

There was no significant difference in fibronectin levels in the epidural tissues between the two treatment groups (Fig. 4). Compared with other treatment groups, the tissue expression of matrix metallopeptidase-9 (MMP-9) was enhanced in the cHA group (Fig. 4B). Quantitative analysis assay indicated that levels of matrix metalloproteinase-3 (MMP-3), and 9 levels were significantly up-regulated in the epidural tissues of the cHA groups (Fig. 4C).

### cHA suppressed the expression of TGF-*β*1 and vimentin, but enhanced the expression of CD44 and IL-6

**Figure 5:**
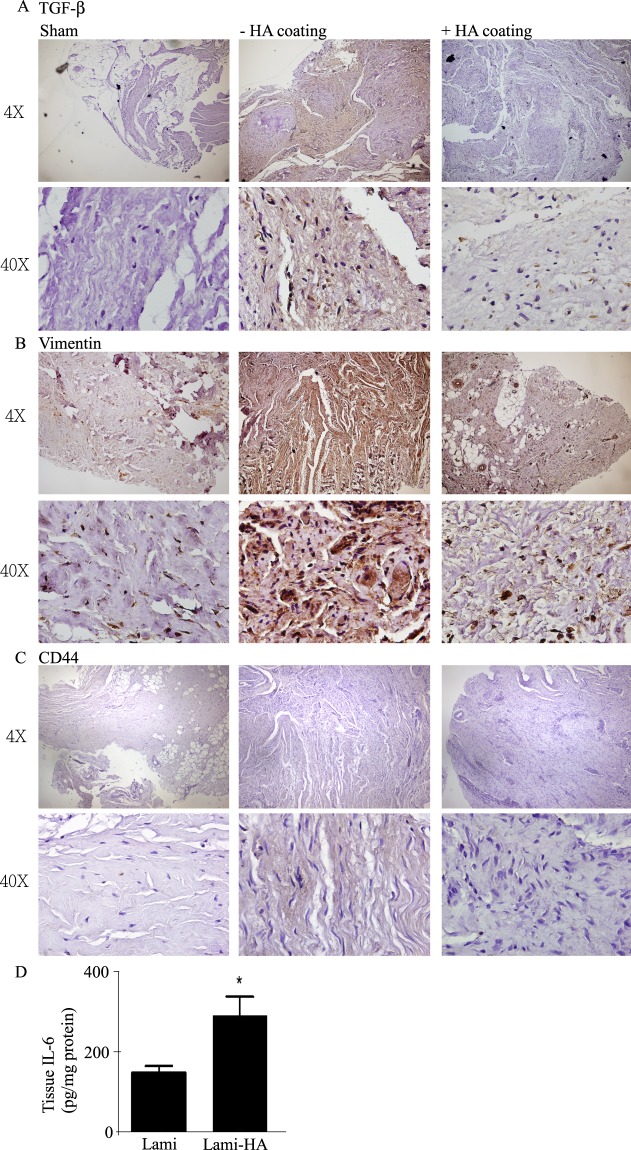
(**A**) TGF-*β*1 expression was decreased in the cHA group. (**B**) Vimentin expression was decreased in the cHA group. (**C**) CD44 expression was enhanced in the cHA group. (**D**) The level of IL-6 was significantly increased in the cHA group in comparison with the non-cHA group.

The increased expressions of transforming growth factor beta 1 (TGF-*β*1) and vimentin in the epidural tissues were restored to the baseline levels following treatment with cHA (Fig. 5), indicating the attenuation of epithelial mesenchymal transition (EMT). The expressions of CD44 and tissue levels of interleukin-6 (IL-6) were augmented in the epidural tissue of cHA group (Fig. 5C,D).

## Discussion

EF is a regenerative process that can lead to failed back surgery syndrome following spinal laminectomy. It was hypothesized that 1,4-butanediol diglycidyl ether-crosslinked hyaluronan would demonstrate inhibitory effects on EF through the effects of MMPs-mediated ECM remodeling and anti- inflammatory pathways. The results of the study supported our hypothesis and showed that treatment with cHA improved the organization of ECM components during the cellular healing process.

It was found that cHA application significantly enhanced MMP-3 and MMP-9 expression in the scar tissues. TGF-*β*1 and vimentin levels in tissue were suppressed and anti-inflammatory cytokines (CD44 and IL-6) were expressed at higher levels in the cHA group. To our knowledge this is the first experimental study that has demonstrated locally applied cHA can significantly inhibit EF formation following hemi-laminotomy and laminectomy in pigs. More specifically, the anti-fibrotic effect of cHA following hemi-laminotomy and laminectomy in pigs could be affected by the enhancements in MMPs expression and anti-inflammatory pathways.

cHA hydrogel is a safe, biocompatible, and nontoxic material. It is also effective in reducing adhesions^6,8^. Unlike traditional HA, cHA is highly viscous and can readily coat to the surface of laminectomy defects and adhere to tissue even when the surface is vertical before it degrades. In this study, the MRI study and H&E staining were performed to examine the changes following the surgery. MRI data showed that cHA application prevented EF. H&E staining showed the cHA treated group had better organization of the ECM in the remodeled tissue with reduced infiltration of inflammatory cells which was similar to the control groups.

Although the well-organized ECM without infiltration of immune cells was noted through H&E staining in the cHA group, the underlying mechanism remains unclear. The current results suggest that the wound healing process may be mediated through increased cellular proliferation and the suppression of cell death. Healing damaged tissue begins with cell proliferation, and Ki-67 is an important marker for cell proliferation^18,19^. Examining cell death is another method to assess the status of a cell. Several pathways can lead to cell death, such as programmed cellular death (apoptosis) and necrosis. Apoptosis is a response to stressful stimuli in which a cascade is initiated to control cell damage. PARP, which indicates DNA fragmentation, is the final step of apoptosis^20^. Previous studies have shown that cHA could be the ligand targeted to the receptor to activate the downstream signaling in order to maintain the ECM structure and prevent the cell death^6,21^. Since cHA seems to sustain in tissues by Alcian blue staining, we thought that the reduction of epidural fibrosis may be related to enhanced cellular proliferation (increased Ki-67) and reduced apoptosis (decreased PARP).

One of the characteristics of fibrosis is excess deposition of a collagen-rich ECM. Interstitial collagen is the primary component of fibrotic lesions, and the excess deposition of which can result in disruption of tissue structure and function^22^. Meanwhile, increased expression levels of MMPs leads to reductions in ECM formation, and in turn, decreased fibrosis. In this study, it was found that cHA inhibited spinal epidural fibrosis and elevated the levels of MMP-3 and MMP-9. Accordingly, the probability of cHA impeding fibrosis through the modulation of ECM components is high. In addition, other studies have noted that the activation of MMPs expression is directly influenced by cytokines^17,23^. We found that cHA increased cytokine IL-6 production in scar tissue. It is possible that cytokine over-production is associated with the activation of MMPs, which facilitated the anti-fibrotic effect of cHA. However, further investigation will be needed to draw more definite conclusions.

The EMT pathway is central to the wound repair system. Prior research has shown that the inflammatory cytokine TGF-*β* and transcription factor Vimentin activate epithelial mesenchymal transition, which in turn, promotes cellular transformation into mesenchymal type^24–26^. TGF-*β* is an inflammatory cytokine that is upregulated during the acute phase of tissue damage, and is crucial to the immune response and wound repair process^27,28^. The surgical interventions in this study acted as simulations of the stress caused by tissue damaged and activated wound repair and immune responses. It has been shown that HA can be used to prevent inflammation as well as fibrosis^29^. Our results showed that cHA application could suppress the release of inflammatory cytokines and Vimentin, an EMT-related marker. At the start of inflammation, TGF-*β* activates inflammatory cytokines and promotes fibrosis in a positive feedback loop. The TGF-*β* ligand then binds to its receptor; and in doing so, the SMAD and EMT pathways are activated, including Vimentin activation^30–32^. Our results are identical to the present signaling pathway and demonstrate that cHA can be used in the spinal surgery with benefit to suppress TGF-*β* secretion, and furthermore, to suppress fibrosis. Meanwhile, we suggest that cHA can act through CD44 binding to regulate the downstream signaling pathway. CD44 is a transmembrane glycoprotein and a primary HA-binding protein. CD44 is also commonly secreted in T cells, monocytes, granulocytes, and fibroblasts^33^. The CD44 receptor is activated by HA and the downstream signaling mediates anti-inflammatory responses^34,35^. Extrapolating from the previous two points, although surgical interventions will induce inflammatory responses through the EMT pathway, anti-inflammatory effects can be observed with the topical application of cHA; cHA suppressing the release of inflammatory cytokines and tissue fibrosis formation through CD44.

The results of this study must be interpreted in light of some limitations. Firstly, despite the well-known comparative rating system and histology analysis selected for the evaluation of the growth of fibrosis, more comprehensive operation-area calculations and histopathological examinations would have improved the objectivity of this work. Another point to consider is that prior research into HA has shown that the migration of lymphocytes, macrophages, and granulocytes is inhibited^36^. Moreover, cHA impedes cytokine formation, thereby accounting for the curtailed inflammatory reactions^37^. However, only the anti-inflammatory effects of cHA through CD44 were able to be recognized in this study. In addition, the selection of L1 as the control level might affect the outcome as biomechanical effects may be different in the upper and the lower lumbar spine.

We demonstrated that the application of cHA in the hemi-laminotomy and laminectomy procedures decreases the extent of epidural fibrosis in swine models. cHA may be a novel biomaterial to prevent epidural fibrosis. In the future, cHA may not only be a filler or cosmetic application in the clinical setting but can be an effective biomaterial in prevention of tissue fibrosis in spinal operations. However, the exact molecular mechanisms underlying cHA remain unclear and so further investigations are warranted to draw further conclusions.

## Material and Methods

### Ethics statement

Six male Lanyu Small-Ear Pigs weighing 20 to 25 kg were obtained from the Taitung Animal Propagation Station (Taitung, Taiwan) and housed in the Laboratory Animal Center of our university. The pigs were housed individually in a room with a 12-h light/dark cycle and central air conditioning (25 °C, 70% humidity). All animal experimental procedures were approved by the Institutional Animal Care and Use Committee (IACUC; National Cheng Kung University Hospital, Taiwan) (IACUC Approval No.:103325) and were performed in accordance with the Guide and Use of Laboratory Animals (Institute of Laboratory Animal Resources).

### Materials

The cross-linked HA hydrogel was prepared by crosslinking HA with 1,4-butanediol diglycidyl ether in a water plus ethanol solution (Maxigen Biotech Inc., New Taipei City, Taiwan) containing no free HA. This study used cHA rather than HA as previous studies have shown cHA has better mechanical stability, viscoelastic properties, and uniformity than HA^8,10^.

### Surgical procedures and groups

Pigs were premedicated with a Telezol/xylazine mixture (3–6 mg/kg, i.m.; Dodge Animal Health, Auckland, New Zealand). The animals were then intubated using an endotracheal tube following intravenous administration of pentobarbital (65 mg/kg). General anesthesia was maintained by volatile isoflurane (1.5–2% v/v in oxygen).

The anesthetized-animals were placed in the prone position, and the lumbar region from L1 to L5 was shaved and prepared with an antiseptic. After appropriate surgical draping, a midline incision from L1 to L5 was made, and consecutive lamina and spines were exposed by subperiosteal dissection. L1 was assigned as the control group. A hemi-laminotomy was performed at L2 (Hemi group) and L3 (Hemi-HA group), and a laminectomy was performed at L4 (Lami group) and L5 (Lami-HA group) by removing the spinous process and part of the lamina. cHA was applied on the surfaces of L3 (Hemi-HA group) and L5 (Lami-HA group) regions. The wounds were then closed in layers. After recovery from anesthesia, the animals were returned to their housing and observed daily for injuries or neurologic complications. The piglets were euthanatized and the epidural scar tissues were collected 4 months after surgery.

### Magnetic Resonance Imaging (MRI) evaluation

The formation of epidural fibrosis was quantified by MRI, as previously described^38,39^. Five contiguous axial slices centered on the surgical site were observed. The spinal canal was divided into four quadrants by drawing perpendicular lines from the center of the dural sac. The four quadrants included the right and left anterior epidural spaces, which encompassed the lateral recesses and spinal nerve roots, and the right and left posterior epidural spaces. At the laminectomy level, the posterior border was defined by drawing a line along the posterior bony remnants. The amount of epidural fibrosis present in each quadrant for all imaging slices at the levels of interest (L1-L5) was graded on the following scale of 0–4: grade 0 (no scarring or traces of a scarring); grade 1 (0% to 25% of the quadrant showing scarring); grade 2 (25% to 50% of the quadrant showing scarring); grade 3 (50% to 75% of the quadrant showing scarring); and grade 4 (75% to 100% of the quadrant being showing scarring). We obtained 20 scores in each operative level which encompassed 5 imaging slices with 4 quadrants in each slice.

### Histologic examination

Specimens were fixed in 10% formalin for 48 hours and embedded into a paraffin block. The paraffin-embedded tissue samples were cut in 4-μm -thick sections on a microtome. The selected sections were stained with Hematoxylin and Eosin (H&E), Masson’s trichrome kit (HT15, Sigma-Aldrich), and Alcian blue, and then examined under a light microscope. H&E staining was used to evaluate the morphology and extracellular matrix (ECM)/elastin degradation (identified as loss, fragmentation and disorganization). Alcian blue staining was used to analyze HA expression in the ECM. Masson’s trichrome staining was used for collagen detection and evaluation of the degrees of epidural fibrosis.

### Immunohistochemistry (IHC)

Tissues were embedded in paraffin and sliced for IHC experiment to verify the protein expression. The antibodies against Ki67 (550609, 1:50, BD Biosciences); PARP (ab32064, 1:100, Abcam); fibronectin (GTX61206, 1:250, GeneTex); MMP-9 (10375-2-AP, 1:250, Proteintech); TGF-*β* (ab190503, 1:100, Abcam); vimentin (550513, 1:50, BD Biosciences) and CD44 (GTX83114, 1:500, GeneTex) were used.

### Measuring MMPs levels

MMP-3 and MMP-9 levels were quantified by using the commercially available ELISA kits (GA-E0339PC and GA-E0339PC, GenAsia, respectively) and operated according to the manufactory’s protocols.

### Measuring IL-6 levels

IL-6 levels were determined by using the ELISA kits (Duo-Set; R&D Systems Inc., Minneapolis, MN). Briefly, samples were incubated with biotinylated rabbit antibody for 2 h, and then streptavidine-conjugated horseradish peroxidase was added. The peroxidase reaction was initiated by adding, 3′,5,5′- tetramethylbenzidine/H_2_O_2_ (R&D Systems) for 30 min, and then stopped by adding 0.5 M H_2_SO_4_. The absorbance was measured at 450 nm.

### Statistical analysis

Data presented as mean ± standard deviation (SD). One-way analysis of variance (ANOVA) followed by Student’s t-test were used for pairwise comparisons between treatments. Chi-squire analysis was used for pairwise comparisons between treatments for the data from MRI scans. Significance was set at p < 0.05.
